# Effects of intensive home treatment: a quasi experimental study

**DOI:** 10.1192/j.eurpsy.2026.10392

**Published:** 2026-07-31

**Authors:** N. Mulder, R. Winter, R. van Asperen

**Affiliations:** 1 Psychiatry, Erasmus MC; 2 Parnassia Psychiatric Institute, Rotterdam; 3 GGZ Rivierduinen, Amsterdam, Netherlands

## Abstract

**Introduction:**

Although Intensive Home Treatment (IHT) is intended to prevent hospital admissions, little is known about its effects in a specific population of patients who are already in outpatient treatment (Flexible Assertive Community Treatment; FACT) before the were referred to IHT.

**Objectives:**

To study the effects of psychiatric crisis-care using IHT versus psychiatric crisis-care in Flexible Assertive Treatment (FACT) Teams.

**Methods:**

We used a quasi experimental design to study the effects after 6 months on admissions and Length of Hospital Stay (LOS) of crisis-care in IHT versus FACT. Patients in a crisis situation, who were treated in FACT, were either admitted to IHT or stayed in FACT, based on the treatment availability in IHT.

**Results:**

463 patients in a psychiatric crisis situation were referred to IHT, and 59 patients in a psychiatric crisis situation could not be referred to IHT. After 6-month follow-up 27.2% vs. 33.9% patients were voluntary admitted (a difference of 6,7%; 95% CI: -6.6% to 20.0%), 7.1% vs. 8,5% patients were involuntary admitted (a difference of 1.4%; CI: -6.4% - 9.2), and the median LOS (days) was 31.0 vs. 61.5 in patients who were initially referred to IHT versus those who remained in FACT and could not be referred to IHT.

**Conclusions:**

IHT may thus reduce involuntary admissions as well as hospital-bed days for patients in a psychiatric crisis situation, and referred by outpatient mental health services. The effect on voluntary admissions did not reach statistical significance.

**Disclosure of Interest:**

None Declared

